# Supplementary material for: How do i bite thee? let me count the ways: Exploring the implications of individual biting habits of Aedes aegypti for dengue transmission
Source: PLoS Negl Trop Dis. 2022 Oct 4;16(10):e0010818. doi: 10.1371/journal.pntd.0010818 (PMC9565401; doi:10.1371/journal.pntd.0010818)
Supplement: S1 Text — Fig A: Kolmogorov-Smirnov test to determine whether there was a difference in distributions of the total number of bites at any of the tested temperatures between biological replicates. Significant difference at 24°C only (p = 0.04266). No significant difference was found at 28°C (p = .06322) or 32°C (p = 0.6325). Fig B: We also saw no significant difference in the distribution of time to first bite (Kolmogorov-Smirnov test) at either 24°C (p = 0.3323), 28°C (p = 0.8038), or 32°C (p = 0.2897). Fig C: Similarly, there was no significant difference between biological replicates in the time between first and second bites (Kolmogorov-Smirnov test) at either 24°C (p = 0.0523), 28°C (p = 0.1619), or 32°C (p = 0.8562). Fig D: There was a significant difference between biological replicates in the time between first and last bites (Kolmogorov-Smirnov test) at 24°C (p = 0.04951) and for 28°C (p = 0.03067), but not 32°C (p = 0.6152). Fig E: A schematic of a hypothetical mosquito with an individual bite profile where a bite occurs when M{bite} = 1. Infectious contact from the index case (PH0) at Time = 2 results in the mosquito becoming exposed (M{status} = 1). A bite from the M{status} = 1 mosquito at Time = 5 did not result in transmission to a susceptible individual (SH) as the EIP had not concluded. However, after the EIP, the mosquito status changes to infectious (M{status} = 2) and bites from the mosquito, such as Time = 12 to SH results in a transmission event and changes the status to SH as “unavailable for infection”. Fig F: Local sensitivity analysis for the extrinsic incubation period of DENV in the mosquito vector: The proportion of simulations (y-axis) where a particular mosquito (x-axis) becomes exposed (P(IC—Ms)) or transmits (P(M->H)) per a range of extrinsic incubation periods. Only mosquitoes with at least one scenario resulting in a non-zero probability of transmission are shown. Fig G: Local sensitivity analysis for infectious period of the index case: The [file pntd.0010818.s001.docx]

**How do I bite thee?**

**Christofferson, et al.**

**Supplemental Information**

**Fig A:** Kolmogorov-Smirnov test to determine whether there was a difference in distributions of the total number of bites at any of the tested temperatures between biological replicates. Significant difference at 24°C only (p = 0.04266). No significant difference was found at 28°C (p = .06322) or 32°C (p = 0.6325).


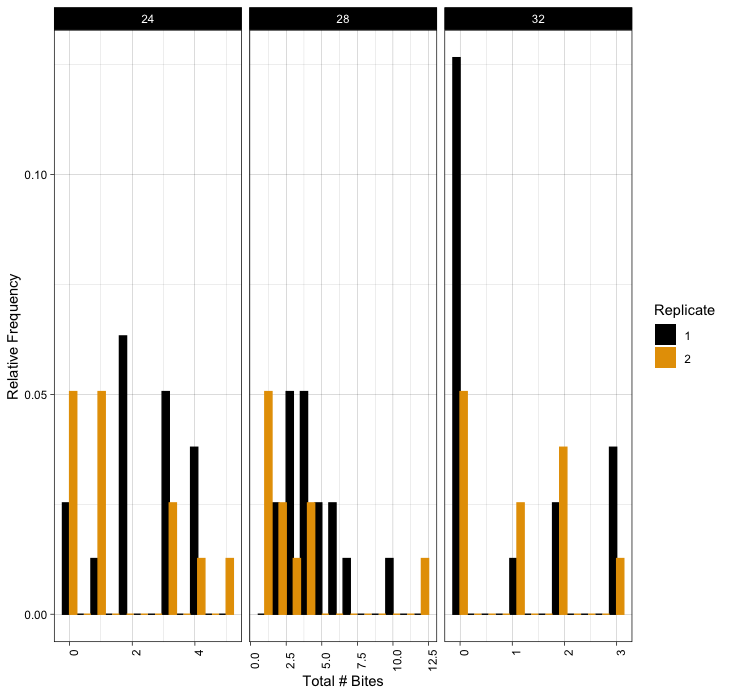


**Fig B:** We also saw no significant difference in the distribution of time to first bite (Kolmogorov-Smirnov test) at either 24°C (p = 0.3323), 28 °C (p = 0.8038), or 32°C (p = 0.2897).


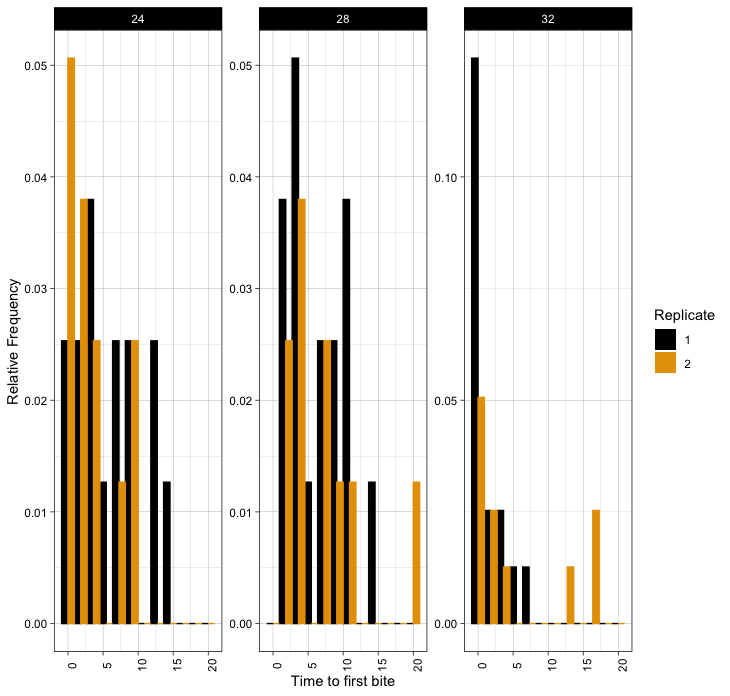


**Fig C:** Similarly, there was no significant difference between biological replicates in the time between first and second bites (Kolmogorov-Smirnov test) at either 24°C (p = 0.0523), 28°C (p = 0.1619), or 32°C (p = 0.8562).


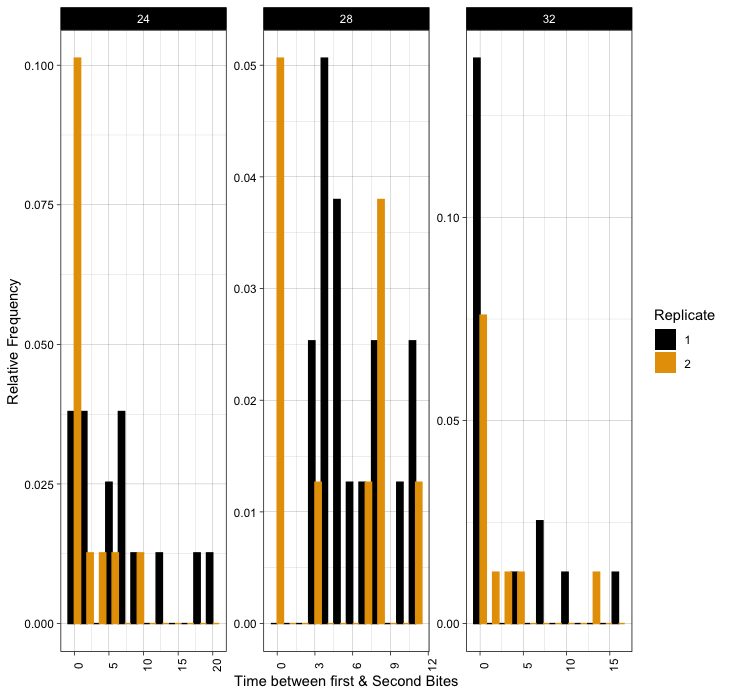


**Fig D:** There was a significant difference between biological replicates in the time between first and last bites (Kolmogorov-Smirnov test) at 24°C (p = 0.04951) and for 28°C (p = 0.03067), but not 32°C (p = 0.6152).


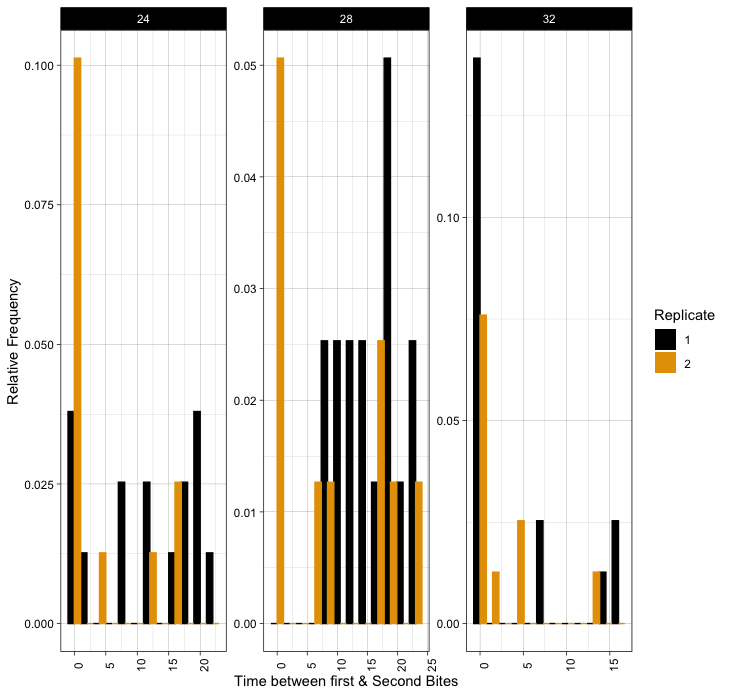


**Fig E**: A schematic of a hypothetical mosquito with an individual bite profile where a bite occurs when M{bite} = 1. Infectious contact from the index case (P_H0_) at Time = 2 results in the mosquito becoming exposed (M{status} = 1). A bite from the M{status} = 1 mosquito at Time = 5 did not result in transmission to a susceptible individual (S_H_) as the EIP had not concluded. However, after the EIP, the mosquito status changes to infectious (M{status} = 2) and bites from the mosquito, such as Time = 12 to S_H_ results in a transmission event and changes the status to S_H_ as “unavailable for infection”.


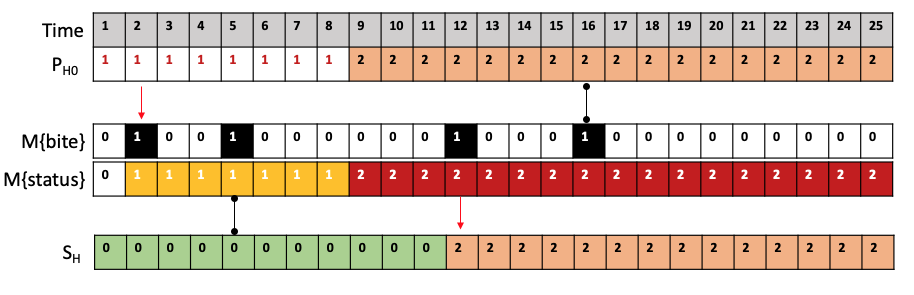


**Supplemental Information: R packages (version) used:** ggplot2 (3.3.6)[1], ggthemes (4.2.4)[2], doBy (4.6.13)[3], dplyr (1.0.9)[4], reshape2 (1.4.4)[5], gridExtra (2.3)[6], EnvStats (2.7.0)[7], ggfortify (0.4.14)[8], corrplot (0.92)[9], ggcorrplot (0.1.3)[10], Hmisc (4.7-0)[11].

**Fig F: Local sensitivity analysis for the extrinsic incubation period of DENV in the mosquito vector:** The proportion of simulations (y-axis) where a particular mosquito (x-axis) becomes exposed (P(IC—Ms)) or transmits (P(M->H)) per a range of extrinsic incubation periods. Only mosquitoes with at least one scenario resulting in a non-zero probability of transmission are shown.

**
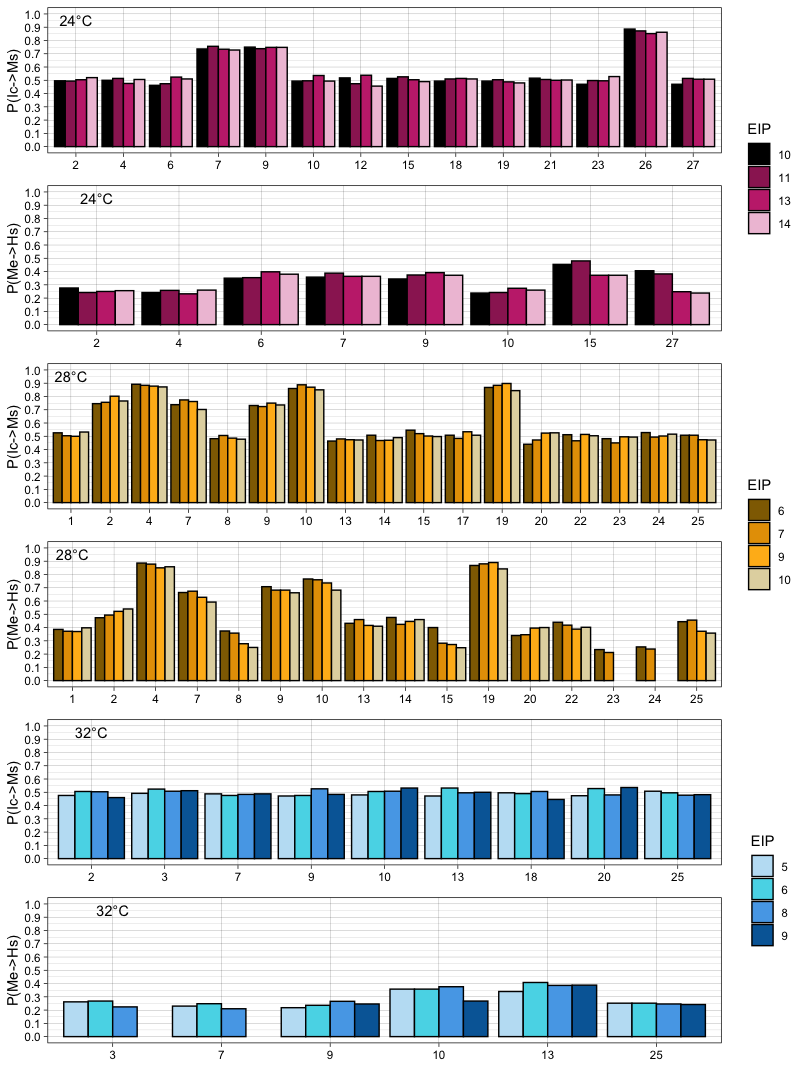
**

**Fig G: Local sensitivity analysis for infectious period of the index case:** The proportion of simulations (y-axis) where a particular mosquito (x-axis) becomes exposed (P(IC—Ms)) or transmits (P(M->H)) per a range of infectious periods. Only mosquitoes with at least one scenario resulting in a non-zero probability of transmission are shown.


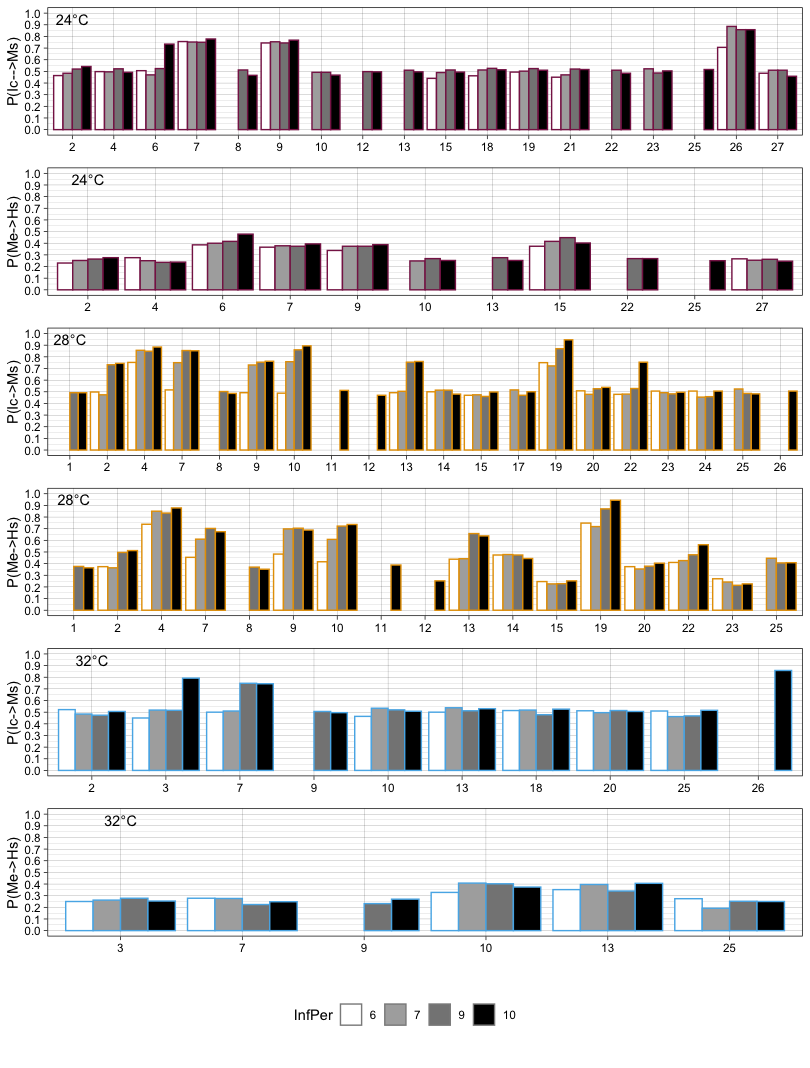


**
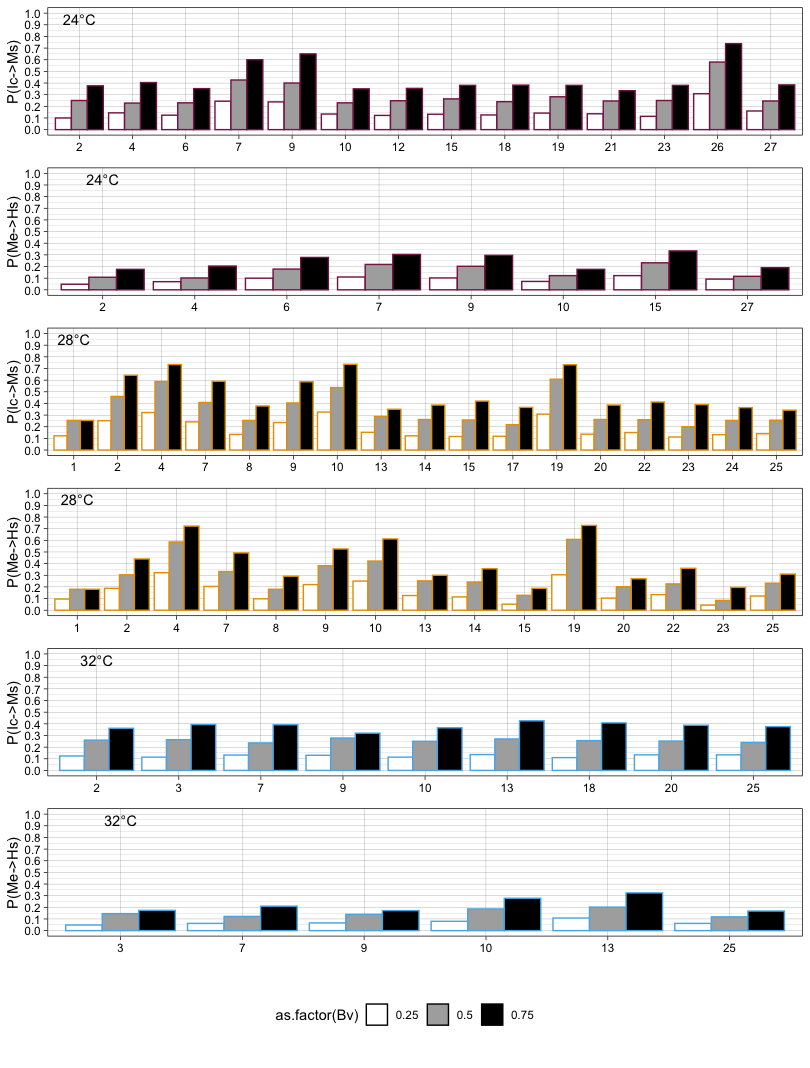
Fig H: Local sensitivity analysis for the probability of transmission given contact between a susceptible mosquito and the infectious index case (𝛽_v_):** The proportion of simulations (y-axis) where a particular mosquito (x-axis) becomes exposed (P(IC—Ms)) or transmits (P(M->H)) per a range of 𝛽_v_. Only mosquitoes with at least one scenario resulting in a non-zero probability of transmission are shown.

**Fig I: Local sensitivity analysis for the probability of transmission given contact between an infectious mosquito and susceptible human (𝛽_h_):** The proportion of simulations (y-axis) where a particular mosquito (x-axis) transmits (P(M->H)) per a range of 𝛽_h_. Only mosquitoes with at least one scenario resulting in a non-zero probability of transmission are shown.

**
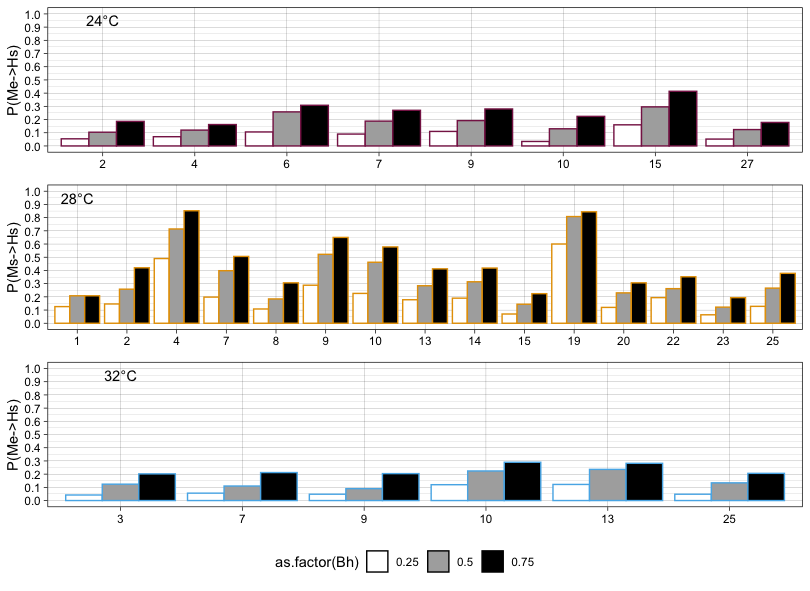
**

**Fig J.** Complete correlation matrices for all three temperatures. X indicate 𝜌 is not significantly different from 0.

**
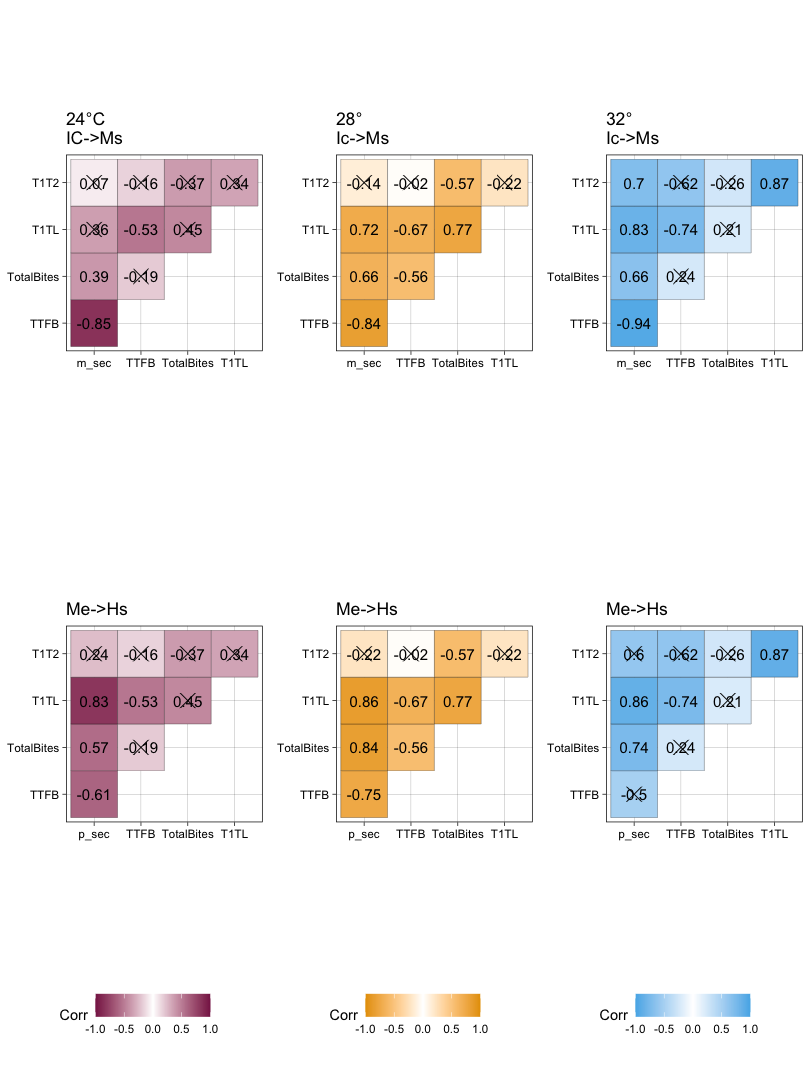
**

**Fig K:** The outcomes of mosquito exposure and subsequent transmission to the susceptible household member derived directly from Equations 1 & 2 (see Methods).

**
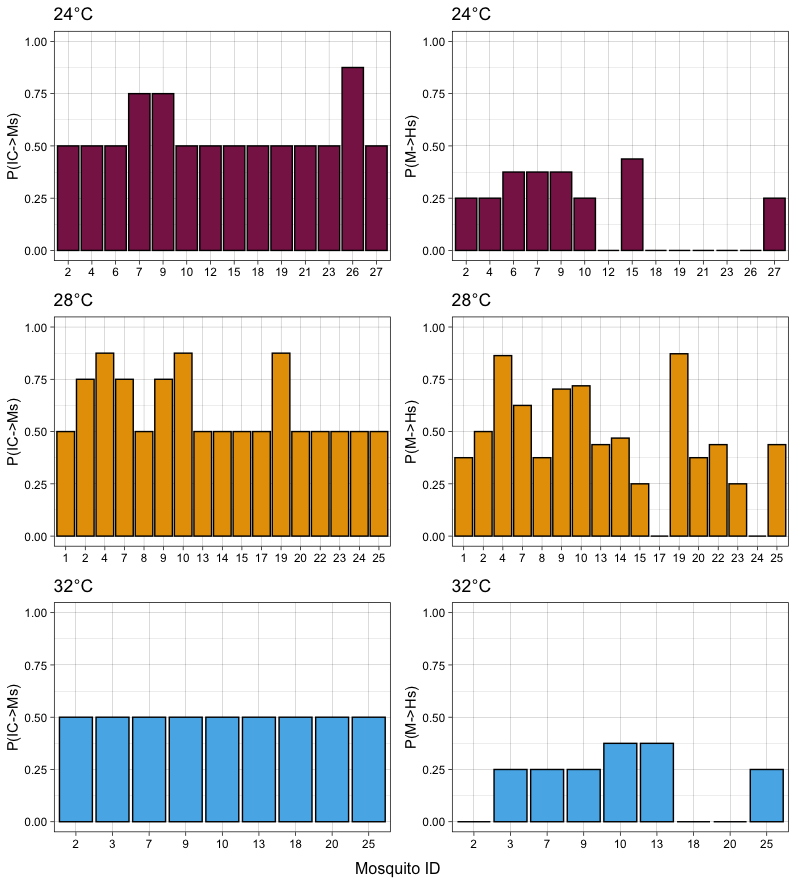
**

**Table A**: Comparison of the proportion of mosquitoes that bit at least once between the experimental data and simulated population at each temperature, using prop.test function in R.

| Temperature | Data Type | Proportion | P-value |
| --- | --- | --- | --- |
| 24 | Experimental | 0.778 | p < .0001 |
|  | Simulated | 0.914 |  |
| 28 | Experimental | 1 | p > 0.05 |
|  | Simulated | 1 |  |
| 32 | Experimental | 0.462 | p < .0001 |
|  | Simulated | 0.914 |  |

**Table B**: Comparison of the proportion of mosquitoes that bit at least twice between the experimental data and simulated population at each temperature, using prop.test function in R.

| Temperature | Data Type | Proportion | P-value |
| --- | --- | --- | --- |
| 24 | Experimental | 0.593 | p < .0001 |
|  | Simulated | 0.914 |  |
| 28 | Experimental | 0.846 | p < .0001 |
|  | Simulated | 0.991 |  |
| 32 | Experimental | 0.346 | p < .0001 |
|  | Simulated | 0.940 |  |

References

1. Wickham, H., W. Chang, and M.H. Wickham, *Package ‘ggplot2’.* Create elegant data visualisations using the grammar of graphics. Version, 2016. **2**(1): p. 1-189.

2. Arnold, J.B. and M.J.B. Arnold, *Package ‘ggthemes’.* 2015.

3. Højsgaard, S., et al., *Package ‘doBy’.* 2022.

4. Mailund, T., *Manipulating data frames: dplyr*, in *R Data Science Quick Reference*. 2019, Springer. p. 109-160.

5. Wickham, H., *Reshaping data with the reshape package.* Journal of statistical software, 2007. **21**: p. 1-20.

6. Auguie, B., A. Antonov, and M.B. Auguie, *Package ‘gridExtra’.* Miscellaneous Functions for “Grid” Graphics, 2017.

7. Millard, S.P. and R. EnvStats-An, *An R package for environmental statistics*. 2013: Springer.

8. Tang, Y., M. Horikoshi, and W. Li, *ggfortify: unified interface to visualize statistical results of popular R packages.* R J., 2016. **8**(2): p. 474.

9. Wei, T., et al., *Package ‘corrplot’.* Statistician, 2017. **56**(316): p. e24.

10. Kassambara, A. and M.A. Kassambara, *Package ‘ggcorrplot’.* R package version 0.1, 2019. **3**(3).

11. Harrell Jr, F.E. and M.F.E. Harrell Jr, *Package ‘hmisc’.* CRAN2018, 2019. **2019**: p. 235-236.
